# Cognitive Impairment Trends Among Older Adults in a Medicaid Home and Community-Based Service Program

**DOI:** 10.1093/geroni/igab046.060

**Published:** 2021-12-17

**Authors:** Richard Fortinsky, Julie Robison, David Steffens, James Grady, Deborah Migneault

**Affiliations:** 1 University of Connecticut School of Medicine, Farmington, Connecticut, United States; 2 University of Connecticut, University of Connecticut, Connecticut, United States; 3 UConn Health, Farmington, Connecticut, United States

## Abstract

Cognitive impairment (CI) is an important risk factor for nursing home admission, but little is known about CI among older adults in Medicaid HCBS programs. Racial and ethnic group CI disparities are found among community-dwelling older adults, but these CI trends have not been explored in Medicaid HCBS populations. In this study, we determined how CI is associated with older adults’ racial and ethnic group identification and educational attainment in Connecticut’s Medicaid HCBS program. The study cohort includes program enrollees age >65 during January-March 2019 (N=3,520). CI measures include: Cognitive Performance Scale (CPS), ranging from 0-8 (cognitively intact to very severe impairment); and a dichotomous measure incorporating Alzheimer’s disease or other dementia diagnosis (ADRD) and CPS score signifying moderate or severe CI. Study cohort characteristics: 75.7% female; age, mean(sd)=79.1(8.2); Non-Hispanic White=47.8%; Non-Hispanic Black=15.9%; Non-Hispanic Other=2.7%; Hispanic=33.6%; HS education=21.7%; mean(sd) CPS score=2.7(1.9); 36.1% with ADRD/high CPS2 score. In multivariate regression models adjusting for age and sex, CPS scores were not independently associated with race and ethnicity, and the likelihood of having ADRD/high CPS scores did not differ by race and ethnicity (all p-values >0.05). In these same models, persons with more than high school education had significantly lower CPS scores (b=-.12; p<.001), and significantly lower likelihood of having ADRD/high CPS scores (AOR=0.61; p<.001), than persons with less than high school education. We conclude that educational level is independently associated with CI, but race and ethnicity are not in this cohort. Policy and practice implications will be discussed.

